# Identification of microRNAs expressed in the midgut of *Aedes albopictus* during dengue infection

**DOI:** 10.1186/s13071-017-1966-2

**Published:** 2017-02-03

**Authors:** Jianxin Su, Chunxiao Li, Yingmei Zhang, Ting Yan, Xiaojuan Zhu, Minghui Zhao, Dan Xing, Yande Dong, Xiaoxia Guo, Tongyan Zhao

**Affiliations:** 1 State Key Laboratory of Pathogen and Biosecurity, Beijing Institute of Microbiology and Epidemiology, Beijing, 100071 People’s Republic of China; 2 Center for Disease Control and Prevention of Guangzhou Military Region, Guangzhou, 510507 People’s Republic of China

**Keywords:** *Aedes albopictus*, MicroRNA (miRNA), Midgut, Dengue virus (DENV)

## Abstract

**Background:**

The midgut is the first barrier to dengue virus (DENV) infections of mosquitoes and therefore is a major bottleneck for the subsequent development of vector competence. However, the molecular mechanisms responsible for this barrier are unknown.

**Results:**

We constructed three small RNA libraries from the midguts of adult *Aedes albopictus* females that had been fed on either sugar solution, an uninfected blood meal, or a blood meal infected with DENV-2, and112 conserved microRNAs represented by 173 miRNA sequences were identified, with 34 novel microRNAs predicted by Mireap, RNAfold and Sfold software. In addition, the expression of aal-miR-1174, aal-miR-2951 and aal-miR-956 was confirmed *via* stem-loop quantitative real-time PCR **(**qRT-PCR). Compared with microRNA expression profiles of mosquitoes that had ingested a regular blood meal, 43 microRNAs were upregulated and 4were downregulated in mosquitoes that had ingested a DENV-2-infected blood meal. Among the differentially expressed microRNAs, miR-1767, miR-276-3p, miR-4448 and miR-4728-5p were verified *via* stem-loop qRT-PCR.

**Conclusions:**

Analyses indicated that the changing patterns in miRNA expression during DENV-2 infection were significant and varied at different time points post infection. Most miRNA were upregulated at 24 h but were downregulated at 48 h post DENV-2 intake. The aal-miR-4728-5p was chosen for an in vitro transient transfection assay, and the results show that this miRNA enhances DENV replication in C6/36 cells. This study provides the first information on microRNAs expressed in the midgut of *Ae. albopictus* and describes species-specific changes in their expression levels following infection by DENV-2.

**Electronic supplementary material:**

The online version of this article (doi:10.1186/s13071-017-1966-2) contains supplementary material, which is available to authorized users.

## Background

The dengue virus (DENV) is one of the most common causes of vector-borne viral disease in tropical and sub-tropical areas [1, 2]. Approximately 50 million dengue infections occur every year, and at least 2.5 billion people are estimated to be at risk of dengue-related diseases worldwide [3]. *Aedes aegypti* L. and *Ae. albopictus *(Skuse) are the principal vectors of the DENV [4]. Because a dengue fever (DF) vaccine has yet to be developed, vector control is currently the only effective means of preventing this disease [3, 5]. However, despite efforts focused on vector control, the global pandemic of DF has increased dramatically in recent decades because of increases in vector and human population densities, which have been accompanied by a sharp increase in more severe manifestations of the disease, and therefore, alternative control strategies are being investigated. Some of these efforts have been made based on the genetic manipulation of insect vectors to modulate characteristics such as vector competence [6–8], but the manipulation of vector competence must be based on extensive knowledge of the molecular factors underlying vector-pathogen interactions.

Vector competence is the intrinsic ability of arthropod vectors to acquire, maintain, and transmit a pathogen. Several barriers exist in mosquitoes that can hinder the infection, dissemination, and transmission of arbovirus, including DENVs [9, 10]. Of these, the midgut is the first barrier to the invasion of pathogens ingested into the alimentary tract. The ability of midgut epithelial cells to resist viral infection is the main factor determining the susceptibility of mosquitoes to arbovirus infections and a key index of vector competence [11, 12]. Currently, the molecular mechanisms underlying the specific binding between viral pathogens and midgut epithelial cells that regulate viral replication are still unclear. This in turn has been an obstacle to research on the molecular mechanisms responsible for the susceptibility of mosquito vectors to DENV. Understanding the molecular mechanisms underlying these differences in vector competence is important in assessing the risks posed by any particular arbovirus, assessing mosquito vector combinations and indeveloping novel strategies to mitigate or block the transmission of mosquito-borne arboviruses.

MicroRNAs (miRNAs) are a class of non-coding RNAs that regulate gene expression at the post-transcriptional level [13, 14]. Several studies have shown that miRNAs play important roles in controlling viral infections and conferring innate immunity [15, 16]. In *Anopheles gambiae*, Dcr1, Dcr2 and Drosha transcripts have been shown to exhibit enhanced associations with polysome in the midgut after *Plasmodium* infection [17], and knocking down Dcr1 and Ago1 mRNAs in the midgut of this species led to an increased susceptibility to *Plasmodium* infections [18]. Transgenic *Ae. aegypti* mosquitoes in which Aa-dcr2 gene expression in the midgut had been suppressed,show significantly higher Sindbis virus titres, midgut infection rates and virus dissemination rates than normal mosquitoes [12]. In *Ae. aegypti*, a transgenic family that expressed an inverted-repeat RNA in the midgut was constructed that displayed a remarkable reduction in DENV-2 replication in the midgut after ingesting blood meals infected with DENV-2 [19]. These experiments demonstrate that miRNAs play important roles in the process of pathogen infection in mosquitoes and can affect the sensitivity of the midgut to DENV infection. They also provide a theoretical basis for the genetic manipulation of vector competence.

Currently, most studies on miRNAs in *Aedes* mosquitoes have focused on *Ae. aegypti*, in which at least 88 miRNAs have been identified (miRBase 20, http://www.mirbase.org/). Several studies have shown that DENV infection causes changes in miRNA expression in the midgut of this species, some of which enhance DENV infections in cultured cell lines [20, 21]. Although *Ae. albopictus* and *Ae. aegypti* both belong to the subgenus *Stegomyia* [22], *Ae. aegypti *is generally a more efficient disease vector but is less susceptible to DENV infections than *Ae. albopictus*. In general, more susceptible species can become infected by a lower viral load than less susceptible species, which suggests that *Ae. albopictus* could also function as a maintenance vector during inter-epidemic periods [23]. Several studies have demonstrated that the midgut of *Ae. albopictus* is more susceptible to DENV infection than that of *Ae. aegypti*, but the subsequent dissemination of DENV from the midgut is slower in *Ae. albopictus* [24–26], thus the miRNA involving in midgut infection by DENV should be different. These results suggest the existence of interspecific differences in both vector competence and the molecular mechanisms responsible for the midgut invasion barrier [27].

Although some *Ae. albopictus* miRNAs have previously been identified, none were midgut miRNAs. Many miRNAs have distinct expression patterns in different organs, and some areeven tissue-specific [28]. For example, miR-1175, miR-1174, miR-281 and miR-989 are only expressed in the midgut of *An. gambiae,* and the expression of miR-989 is restricted in females. MiR-12 and miR-283 are expressed in the gut and thorax of *An. gambiae* and in the foregut, posterior midgut, hindgut and salivary glands of *Drosophila melanogaster* embryos [29]. These tissue-specific expression patterns of some miRNAs imply that they have different roles in different organs. Because the midgut is the first barrier to a DENV infection [11, 12], identifying the patterns and potential roles of different midgut miRNAs during the course of a DENV infection could aid in the identification of the molecular mechanisms responsible for the midgut infection barrier and thereby facilitating the control of DENV-related diseases. Here, we present the first investigation of midgut miRNAs in *Ae. albopictus*, including changes in their expression levels during the process of DENV infection in this species.

## Methods

### Mosquito collection and husbandry

*Aedes albopictus* mosquitoes were collected from Guangzhou City in 2012 and reared in an insectary under laboratory conditions (temperature 26 ± 1°C, relative humidity 80%, light: dark photoperiod 14:10) to the 5th (5F) generation. Adult mosquitoes were provided with a 10% glucose solution, and females were allowed to feed on the blood of healthy mice to produce eggs.

Adult female mosquitoes were randomly assigned to three groups 4 to 6 days after emergence. Group C was fed on sugar solutiononly; Group B was fed on uninfected blood meal (blood:glucose solution:brain suspension of normal suckling mice = 1:1:1); and Group D was fed on an artificial DENV-2 blood meal (blood:10% glucose solution: DENV suspension = 1:1:1). Mosquitoes were starved for 12–16 h before being allowed to feed for approximately 1 h. Fully engorged mosquitoes were selected after cold anaesthetization at -20 °C for 2 min and transferred to a separate insectary under the conditions described above. Midguts were dissected from mosquitoes 24–26 h after they had fed.

### Virus strains

DENV-2 virus (New Guinea C strain, NGC) was obtained from the Microbial Culture Collection Center of the Beijing Institute of Microbiology and Epidemiology (Beijing, China). The DENV-2 starting stock was prepared in 1-day-old suckling mice *via* intracerebral inoculation. A suspension of infected mouse brains was made in Dulbecco’s modified Eagle’s medium (DMEM, Gibco) and stored at -70 °C until use.

### Mosquito dissection and RNA extraction

Midguts from approximately 100 mosquitoes from each of the C, B and D groups were collected by dissecting individual mosquitoes in a drop of cold physiological saline. Midguts from the three groups were stored separately in 1.5-ml RNase-free microcentrifuge tubes with 1.0 ml RNA-preserving liquid and then flash frozen and stored at -80 °C until subsequent RNA isolation. Total RNAs from the three groups were extracted using the RNeasy Mini Kit (Qiagen, Beijing, China) according to the manufacturer’s instructions. The final total RNA was dissolved in 20μl RNase-free water.

Two sets of samples were prepared; one set was stored at -80 °C for future verification, and the other was analysed *via* electrophoresis on a 15% denaturing polyacrylamide gel after which small RNAs in the 15–30 nt range were purified and ligated with a 3' adapter (5PUCGUAUGCCGUCUUCUGCUUGUidT) and a 5' adapter(5GUUCAGAGUUCUACAGUCCGACGAUC). These were then reverse transcribed using the primer 5'-CAA GCA GAA GAC GGC ATA CGA-3' and proliferated using forward and reverse primers (5'-AAT GAT ACG GCG ACC ACC GAC AGG TTC AGA GTT CTA CAG TCC GA-3' and 5'-CAA GCA GAA GAC GGC ATA CGA-3'). PCR products were purified *via* phenol/chloroform extraction and ethanol precipitation and shipped to BGI (Shenzhen, China) for high-throughput deep sequencing.

### qRT-PCR

Stem-loop qRT-PCR analysis was performed using an ABI STEP-ONE PLUS Real Time PCR System (Applied Biosystems). The specific miRNA stem-loop primers were designed by BGI (Shenzhen, China), and the Real-Time primers were designed with Primer2.0. All primers are shown in Table 1. The reverse transcription reaction was performed with the One Step PrimeScript RT Reagent Kit (Qiagen, Beijing, China) according to the manufacturer’s protocol. Three replicates were performed for each sample, and the *Ae. albopictus* house-keeping rpS7 gene was used as an internal reference [30]. The relative expression of each miRNA was calculated using the2^-△△ CT^ method [31]. The reaction mixture contained 1.6 μl MgCl_2_ (TaKaRa), 0.1 μl SYBRGEEN (Invitrogen), 2 μl 10× buffer (TaKaRa), 0.4 μl dNTP (10 mM/each; BGI, China), 0.2 μl forward and reverse primer (50 pM/μl; BGI, China), 1 μl Template (cDNA), 0.1 μl ROX (Invitrogen), 0.2 μl Taq (5U/μl; Kappa,USA) and 14.2 μl H_2_O in a final volume of 20 μl. The qRT-PCR program was 95 °C for 2 min, followed by 40 cycles of 94 °C for 10 s, 53 °C for 10 s and 72 °C for 40 s. The melting curves of selected miRNAs were determined after amplification using the following program: 95 °C for 30 s, and 65 °C for 15 s, followed by an increase in temperature to 95 °C while continuously recording the fluorescent signal.

**Table 1:** Primer sequences used in this study

miRNA name	Primer name	Sequence (5'–3')
aal-miR-1767	RT-primer	GTCGTATCCAGTGCGTGTCGTGGAGTCGGCAATTGCACTGGATACGAC**ACCTTG**
	Forward primer	AGACAGGAGAACAGCA
aal-miR-276-3p	RT-primer	GTCGTATCCAGTGCGTGTCGTGGAGTCGGCAATTGCACTGGATACGAC**GAGCAC**
	Forward primer	TAGGAACTTCATACCG
aal-miR-4448	RT-primer	GTCGTATCCAGTGCGTGTCGTGGAGTCGGCAATTGCACTGGATACGAC**ACCCCT**
	Forward primer	GGCTCGTTGGTCTAGG
aal-miR-4728-5p	RT-primer	GTCGTATCCAGTGCGTGTCGTGGAGTCGGCAATTGCACTGGATACGAC**TGCTGC**
	Forward primer	TGGGAGGGCAGAGGGG
aal-miR-1174	RT-primer	GTCGTATCCAGTGCGTGTCGTGGAGTCGGCAATTGCACTGGATACGAC**AGTTGG**
	Forward primer	TCAGATCTAACTAATACCCAA
aal-miR-2951	RT-primer	GTCGTATCCAGTGCGTGTCGTGGAGTCGGCAATTGCACTGGATACGAC**TCGCCC**
	Forward primer	AAGAGCTCAGCACGCAGG
aal-miR-956-3p	RT-primer	GTCGTATCCAGTGCGTGTCGTGGAGTCGGCAATTGCACTGGATACGAC**AATGAT**
	Forward primer	TTTCGAGACCACTGCAAAT
aal-miR-956-5p	RT-primer	GTCGTATCCAGTGCGTGTCGTGGAGTCGGCAATTGCACTGGATACGAC**AGTTAA**
	Forward primer	GTTTGAAATGGTCTCGTTAAC
Universal	Reverse primer	GTGCGTGTCGTGGAGTC
rpS7	Forward primer	ATGGTTTTCGGATCAAAGGT
	Reverse primer	CGACCTTGTGTTCAATGGTG
DENV-2	Forward primer	TCAATATGCTGAAACGCGCGAGAAACCG
	Reverse primer	TTGCACCAACAGTCAATGTCTTCAGGTTC

The Probe Library probe binding site is shown in bold type

### Alignment of conserved miRNAs using BLAST and tag2 miRNA software

We first mapped all clean small RNA tags by matching them to GenBank rRNA, scRNA, snoRNA, snRNA and tRNA databases and removed matched tags from unannotated tags. To make sure every unique small RNA mapped to only one annotation, we adhered to the following priority rule: all rRNA (in which GenBank > Rfam) > repeat > exon > intron > known miRNA. Because miRBase does not currently contain *Ae. albopictus* miRNA, we first used BLAST to align small RNA tags with the *Ae. aegypti* miRNA precursor in miRBase19.0 to obtain a miRNA count with no mismatches. We then aligned tags to all mature animal miRNAs in miRBase19.0 using tag2 miRNA software (developed by BGI; Shenzhen, China). The specific steps we used were as follows: (i) We first matched mature sequences from our clean data to those of animal miRNAs in miRBase19.0, considering interspecific variations and allowing up to two mismatches and a few gaps; (ii) From the miRNA families identified through the above process, we selected miRNA sequences with the highest expression levels among the different species in the same family for a provisional miRNA database; (iii) We then constructed a miRNA database by comparing and aligning the cleandata to known miRNA expression profiles. We calculated expression levels by comparing the clean data to miRNA sequences in the provisional database and by summing the number of those that aligned with up to 2 mismatches. This verification process gave us a high probability of obtaining meaningful results.

### Prediction of novel miRNA candidates using Mireap software

Mireap software was used to predict novel miRNAs by exploring their secondary structure, Dicer cleavage sites and the minimum free energy of unannotated small RNA tags that could be mapped to the *Ae. aegypti* genome*.* Mireap can be accessed from the following link: http://sourceforge.net/projects/mireap/. Some key conditions for novel miRNA prediction are as follows: (i) The tags used to predict novel miRNAs were unannotated tags that could be perfectly matched to the intron and antisense exon regions of the reference genome (*Ae. aegypti*); (ii) Of those genes whose sequences and structures satisfied the above criteria, those with hairpin miRNAs that could enfold secondary structures or mature miRNAs present in one arm of the hairpin precursors were considered as candidate miRNA genes; (iii) Mature miRNA strands and their complementary strand (miRNA*) 2-nucleotide 3’ overhangs and (iv) hairpin precursors lacking large internal loops or bulges were considered; (v) The secondary structures of the hairpins had to bestable, with a minimum free energy (MFE) lower than or equal to -20 kcal/mol; and (vi) the number of mature miRNAs with predicted hairpins must have been no less than 5 after alignment.

RNAfold (http://rna.tbi.univie.ac.at/cgi-bin/RNAfold.cgi) was used to screen novel miRNA candidates that satisfied criteria ii-v mentioned above. For those structures predicted by RNA fold for which MFE was > -25, Sfold (http://sfold.wadsworth.org/cgi-bin/srna.pl) was used to determine if these were novel miRNAs according to criteria ii–v above.

### Analysis of variations in miRNA expression

To compare the miRNA expression levels in groups C, B and D *via* high through put deep sequencing, we normalized the read numbers in each library according to the following formula: $\mathrm{Normalized}\ \mathrm{expression}\kern0.74em =\frac{\mathrm{Actual}\ \mathrm{miRNA}\ \mathrm{reads}}{\mathrm{Total}\ \mathrm{count}\ \mathrm{of}\ \mathrm{clean}\ \mathrm{reads}}\times {10}^6.$ We then calculated the ratio and the magnitude of between-group differences and their associated *P*-values from the normalized data. The ratio was calculated according to the following formula: $\mathrm{ratio}\kern0.5em =\kern0.5em \frac{\mathrm{normalized}\ \mathrm{expression}\ \mathrm{of}\ \mathrm{treatment}\ \mathrm{group}}{\mathrm{normalized}\ \mathrm{expression}\ \mathrm{of}\ \mathrm{control}\ \mathrm{group}}$, and the magnitude of differences is expressed as “fold-change”, which was calculated using the following the formula: fold-change = log_2_ratio. A fold-change > 1 or < -1 with a *P*-value < 0.05 was regarded as being significantly different [32]. Fold-changes and their associated *P*-values were calculated using a special procedure developed by the BGI biotech company (Shenzhen, China).

### Cell culture and cell infection

*Aedes albopictus* C6/36 cells were cultured in RPIM 1640 (Gibco) culture medium supplemented with 10% heat-inactivated foetalbovine serum (FBS, Gibco) and maintained at 28 °C without CO_2_. To establish DENV-2 infections, C6/36 cells were seeded in 12-well plates to a density of approximately 80%. DENV-2 (NGC) at a 0.01 multiplicity of infection (MOI) was diluted in the above growth medium and added to the cells. After being rocked at room temperature for 1 h, the plates were incubated at 37 °C for 72 h.

### Transient transfection of miRNA oligonucleotides

C6/36 cells were transfected with 50 nmol of an aal-mir-4728-5p mimic or inhibitor and negative controls for the mimic (NCm) and inhibitor (NCi) with Lipofectamine 2000 (Invitrogen) according to the manufacturer’s recommendation. The cells were inoculated with the DENV-2 inoculum 24 h post-transfection. qRT-PCR analysis was used to detect the expression levels of aal-mir-4728-5p in C6/36 cells 24 h and 72 h post-transfection and to detect the DENV-2 levels in all C6/36 cells 72 h post-inoculation. The oligonucleotides used in this study are listed in Table 2. All miRNA oligonucleotides were purchased from Genepharma (Shanghai, China).

**Table 2:** Oligonucleotide sequences used in this study

Name	Sense (5'–3')	Antisense (5'–3')
aal-mir-4728-5p mimics	UGGGAGGGCAGAGGGGCAGCA	CUGCCCCUCUGCCCUCCCAUU
Negative control for mimic (NCm)	UUCUCCGAACGUGUCACGUTT	ACGUGACACGUUCGGAGAATT
aal-mir-4728-5p inhitior	UGCUGCCCCUCUGCCCUCCCA	
Negative control for inhibitor (NCi)	CAGUACUUUUGUGUAGUACAA	

### rpS7 q-RTPCR

The *Ae. albopictus* house-keeping rpS7 gene was used as an internal control for the qRT-PCR results. The forward primer sequence was 5'-ATG GTT TTC GGA TCA AAG GT-3', and the reverse sequence was 5'-CGA CCT TGT GTT CAA TGG TG-3'. qRT-PCR analyses followed the protocols described above.

### Statistical analysis

A Poisson distribution was used to analyse adigital transcript of the profile data following the method described by Audic & Claverie [32]. The 2^-△△ CT^ method was used to determine relative expression levels from the qRT-PCR results, and paired *t*-tests were used to determine if the differences were statistically significance using SPSS19.0.

## Results

### Overview of the dataset

**Fig. 1:**
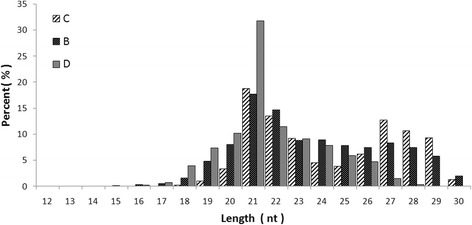
Frequency of the length of small RNAs in the midguts of *Aedes albopictus* mosquitoes fed on sugar solution (C), non-infected blood-meal (B) and blood-meal infected with DENV-2 (D)

To identify miRNAs expressed in the midgut of *Ae. albopictus* and to explore their functions following the ingestion of DENV-infected blood, three small libraries of *Ae. albopictus* midgut RNAs were constructed, one from mosquitoes that had been fed on sugar solution (C), one from those that had been fed on uninfected blood (B) and one from those that had been fed on blood containing DENV-2 (D). All three libraries were sequenced *via* high throughput sequencing, giving totals of 19,709,476, 14,667,037 and 16,085,156 reads from the three groups, respectively. When the three libraries were combined, total read numbers for different lengths of RNA peaked at approximately 21 nt. The distribution of 21-nt sRNAs in C, B and D was approximately 18.81, 16.78 and 31.8%, respectively (Fig. 1). A group of 27-nt clean reads also accounted for a relatively high proportion (16.28, 8.38 and 4.17%, respectively) of the total RNAs in the three libraries; we suspect that these could be piwi-interacting RNAs (piRNAs).

After removal of the adaptor, insert, poly (A) tail and short RNAs < 18 nt, 19,444,780, 14,376,865 and 15,762,225 clean reads were obtained from the C, B and D libraries, respectively. Among the clean reads, miRNA reads accounted for 23.9, 9.17 and 12.25% of the total, and unannotated, redundant reads accounted for 67.36, 47.72 and 51.57% of the C, B and D libraries, respectively.

Because a complete *Ae. albopictus* genome is currently unavailable, we compared the reads obtained to the published *Ae. aegypti* genome (www.vectorbase.org). Although *Ae. albopictus* and *Ae. aegypti* belong to the same subgenus, only approximately 5–8% of the RNAs in the three libraries matched that of *Ae. aegypti* (Table 3). Therefore, the majority of the RNAs in each library remain unidentified.

**Table 3:** Numbers of unique and total sRNAs in the midguts of *Ae. albopictus* that matched to the *Ae. aegypti* genome

Group	Unique sRNAs	Number matched (%)	Total sRNAs	Number matched (%)
C	1,555,751	74,170 (4.77)	19,444,780	5,295,808 (27.24)
B	1,187,330	92,588 (7.8)	14,376,865	5,321,300 (37.01)
D	1,159,505	87,572 (7.55)	15,762,225	6,044,644 (38.35)

Group C was fed on a sugar solution, B was fed on uninfected blood and D was fed on DENV-2 blood

### Conserved and novel miRNAs in the midgut of *Ae. albopictus*

**Fig. 2:**
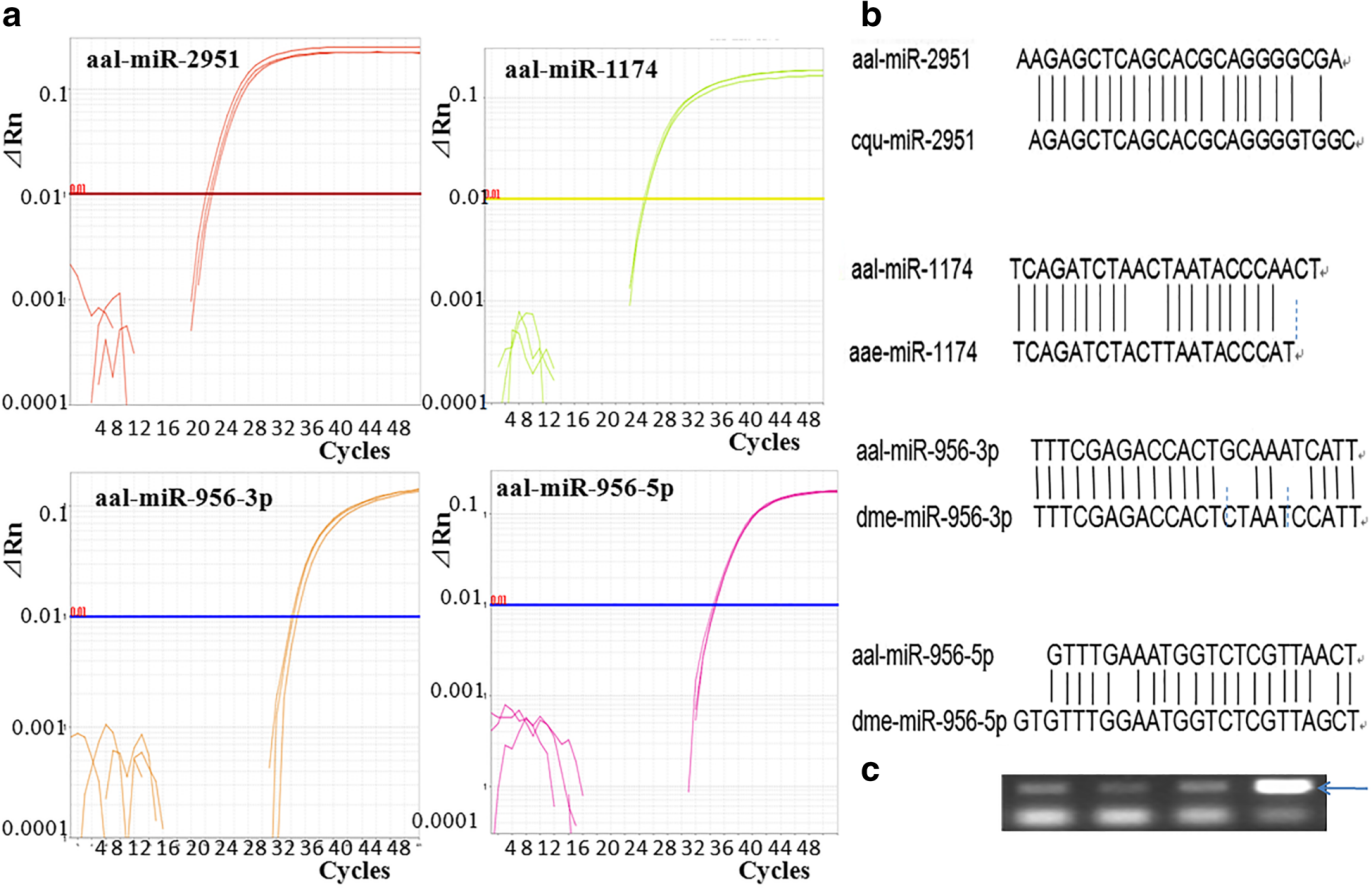
Verification of four miRNAs in the midgut of *Aedes albopictus via* stem loop qRT-PCR. **a** A mplification plots of each miRNA from stem loop qRT-PCR results. **b** A diagram showing the similarity of each of the four miRNAs to conserved, mature miRNA sequences in miRBase19.0. **c** A photograph of the electrophoresis gel (4% agarose) with arrows indicating the size of the expected PCR products corresponding to these four miRNAs

By comparing the three libraries with miRBase19.0, we identified 112 conserved miRNAs represented by 173 miRNA sequences (Additional file 1: Table S1), the majority of which are conserved in other insects, such as *D. melanogaster* and *An. gambiae,* and 71 of these conserved miRNAs had been previously reported in *Ae. aegypti*. However, we did not find five miRNAs that had been previously reported in *Ae. albopictus* [33, 34]: aal-miR-286, aal-miR-309, aal-miR-315, aal-miR-929 and aal-miR-971. Notably, aal-miR-1174, which had not previously been detected in *Ae. albopictus via* northern blotting [35], and aal-miR-2951, which had only previously been reported in *Culex quinquefasciatus* [33] were sequenced in this experiment. Apart from a few mismatches outside the seed region, the sequences of these were perfect matches to cqu-miR-2951 and aae-miR-1174, respectively (miRBase 19.0; Fig. 2b), and their expression was subsequently verified *via* stem-loop qRT-PCR (Fig. 2a, c).

In addition to the conserved miRNAs, a total of 34 novel miRNA candidates were also predicted (Table 4) using Mireap, RNAfold and Sfold. Of these 34 novel miRNA candidates, 2 were expressed after the ingestion of a blood-meal (aal-miR-new33-5p and aal-miR-3960-5p), and the others were expressed before the ingestion of a blood meal.

**Table 4:** Novel predicted miRNA candidates in the midgut of *Ae. albopictus* before and after the ingestion of blood meal using Mireap, RNAfold and Sfold software

Provisional name	Sequence	Length	Supercontig	Start	End	Strand	Mfe
aal-mir-new1-5p	ACGCGGTCGTGCAGGAAATTATT	23	1.1	2,315,096	2,315,175	+	−39.1
aal-mir-new2-5p	TTTTGACACTAGAGCGGGGCC	21	1.103	2,525,153	2,525,242	-	−43.54
aal-mir-new3-3p	TGTTGGAACAGGAGCGGTGACTGG	24	1.1048	247,401	247,496	+	−27.2
aal-mir-new4-3p	ACGTGATCTCCCAGCTGGATT	21	1.104	2,576,089	2,576,167	+	−66.6
aal-mir-new4-5p	TCCAGCTGGGAGATCACGTAC	21	1.104	2,576,089	2,576,167	+	−66.6
aal-mir-new5-3p	TGAATAAGTGCGGTGAAGACA	21	1.1056	241,944	242,019	-	−48.4
aal-mir-new6-5p	TTGAAGGAATCCCTGGAGGCA	21	1.112	2,058,675	2,058,759	-	−32.1
aal-mir-new7-3p	TGGGGTTGGGCGGAAGGGTTG	21	1.1156	159,517	159,603	+	−29.8
aal-mir-new8-5p	ATTCGCACAACAGTCCCATGTT	22	1.125	2,091,162	2,091,244	+	−28.1
aal-mir-new9-3p	AGAATAAAGACTGCGTAGCCA	21	1.127	946,697	946,773	-	−27.7
aal-mir-new10-5p	TTGGCCATTTTGGAACCGGTA	21	1.13	1,968,890	1,968,963	-	−29.7
aal-mir-new11-5p	TTAAACATAACGTCGGAAGTA	21	1.134	1,382,610	1,382,700	-	−47.7
aal-mir-new12-5p	TCTGGAGCAACATTTGAAAAG	21	1.163	418,620	418,704	+	−24.54
aal-mir-new13-5p	GAATTTTGACATTAGAGCGGG	21	1.1642	60,969	61,064	+	−59.1
aal-mir-new14-5p	AGAATTTTGACACTAGAGCAG	21	1.194	1,043,569	1,043,645	-	−32.6
aal-mir-new15-5p	TGAAGGAATCCCTGGAGGCAT	21	1.2	2,052,582	2,052,676	-	−32.1
aal-mir-new16-3p	ATTTTTTTGACTGTAATTTTAT	22	1.245	1,560,997	1,561,095	-	−26.61
aal-mir-new17-5p	GGGAGCGAGATTAAGGCTTGCT	22	1.249	1,089,011	1,089,093	-	−29.81
aal-mir-new18-3p	ATCCCAAGACTGCGTAGCCGT	21	1.292	1,163,558	1,163,655	+	−42.4
aal-mir-new19-5p	CGAATTTTGACACTAGAGCGG	21	1.299	707,787	707,872	-	*−29.5*
aal-mir-new20-3p	GTCCCTCTGGCGCAGCGGATAGCG	24	1.32	579,217	579,298	-	−37.5
aal-mir-new21-3p	TAAGTGCGCTGAAGACATCA	20	1.379	448,733	448,811	+	−51.82
aal-mir-new22-5p	AACGGTCTAGGGTTCATGTCC	21	1.389	711,000	711,091	+	−30.4
aal-mir-new23-5p	AAATTTTGACACTAGAGCGGG	21	1.412	335,537	335,632	-	−48.1
aal-mir-new24-3p	ACGATGAGGATGATGATGGTG	21	1.457	821,927	822,021	+	−31.16
aal-mir-new25-5p	GGGGGAAATCCTGTACGCTGTATG	24	1.51	2,008,649	2,008,731	+	−33.4
aal-mir-new26-5p	TTGGCATAAGGACGTTTGGCA	21	1.526	450,600	450,680	+	−26.7
aal-mir-new27-5p	GAATTTTGACACTAGAGCAGG	21	1.57	59,966	60,061	+	−44.2
aal-mir-new28-3p	CTGAAGAACTTTGCCGAAGAC	21	1.576	275,303	275,389	+	−30.6
aal-mir-new29-5p	ATTAGAATGTGGAATCTGTTTT	22	1.62	576,218	576,309	-	−32.2
aal-mir-new30-5p	TGGGTATTTTCGGAACGGGCT	21	1.64	1,822,262	1,822,351	-	−28.7
aal-mir-new31-3p	CGGAATTCCAACTGATATCCA	21	1.68	2,729,339	2,729,428	-	−35.25
aal-mir-new32-5p	CAATTTTGACACTAGAGCGGG	21	1.784	432,120	432,215	-	−50.9
aal-mir-new33-5p	GGGAGCGAGATTAAGGCTTG	20	1.249	1,089,011	1,089,093	-	−29.81
aal-mir-3960-5p	GGCGGCGGCGGAGGTGGAGGT	21	1.506	579,623	579,702	+	−53.3

*Abbreviation*: *MFE* minimum free energy

### miRNA expression levels in the midgut of sugar-fed *Ae. albopictus*

The normalized data indicate that the expression levels of most miRNAs were low (Additional file 2: Table S2). We divided miRNA sequences in the C group (sugar-fed group) into five classes according to their normalized reads, i.e. < 10; 10–10^2^; 10^2^–10^3^; 10^3^–10^4^; and 10^4^–10^5^, which were designated Class 1 to Class 5. Class 1 (< 10) contained 68miRNAs, Class 2 (10–10^2^) contained 49 miRNAs, Class 3 (10^2^–10^3^) contained 36 miRNAs, and Class 4 (10^3^–10^4^) contained 15 miRNAs that were abundantly expressed, such as miR-317, miR-2940-5p, miR-275-3p, miR-5706. Class 5 (10^4^–10^5^) contained the remaining 5 miRNAs, which were highly expressed in the midgut: miR-956-3p, miR-184, miR-1-3p, miR-34-5p and miR-281-5p. Of these, aal-miR-956-3p was highly expressed in the midgut; aal-miR-956-5p was also sequenced, but its expression level was very low. The sequences of these two miRNAs matched those of dme-miR-956-3p and dme-miR-956-5p, respectively, in *D. melanogaster* (Fig. 2b) and their expression was verified *via* stem-loop qRT-PCR (Fig. 2a, c). MiR-956 was previously only known to occur in *D. melanogaster* [36] in miRBase19.0, and its function remains unknown. The *Ae. albopictus* midgut also shares four of the ten most frequently detected miRNAs in the C7/10 cell line and *Cx. quinquefasciatus* [33], namely, miR-184, miR-317, miR-275 and miR-8.

### Changes in miRNA expression profiles following the ingestion of DENV-infected and non-infected blood

**Fig. 3:**
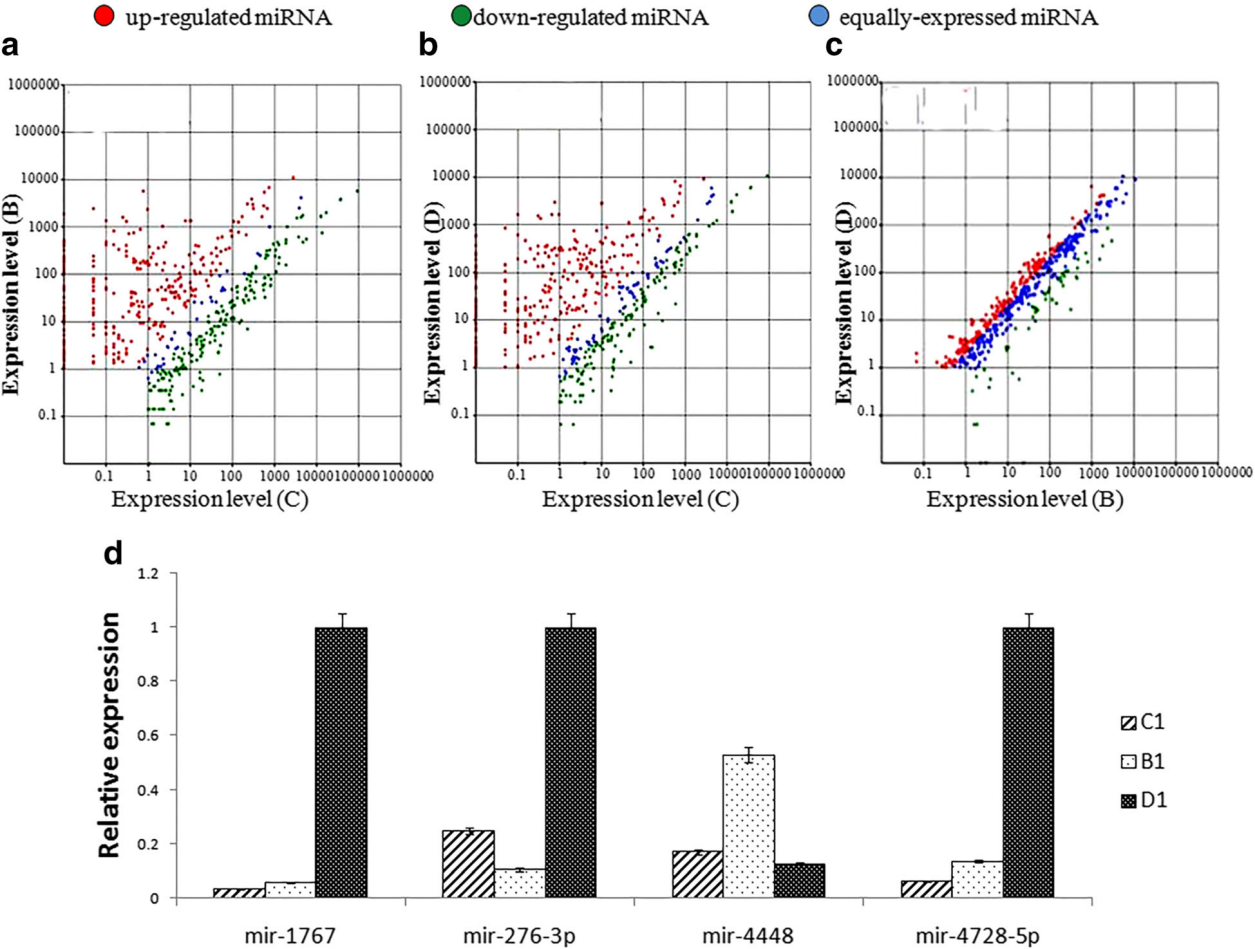
Relative expression of miRNAs in the midguts of *Aedes albopictus* mosquitoes that had been fed either on sugar solution (C), non-infected blood (B) or DENV-infected blood (D). **a** B *vs* C. **b** D *vs* C. **c** D *vs* B. **d** Relative expression of 4 miRNAs in all three groups as determined *via* stem loop qRT-PCR

We compared the normalized abundances of miRNAs by calculating the expression ratio of each miRNA between each of the three treatment groups (Fig. 3a-c). Significant differences in miRNA expression were apparent between all three groups, especially in Groups B and D compared with Group C (Fig. 3a, b). On the basis of the fold-changes and the associated *P*-values calculated for each miRNA, 96 miRNAs were downregulated, and 30 were upregulated in GroupB when compared with Group C. Most of the miRNAs that were highly expressed in Group C, such as miR-956-3p, miR-184, miR-1, miR-34, miR-281, miR-317, let-7, miR-2945, miR-8, miR-71, miR-275, miR-8, miR-1174, miR-1175, miR-989, miR-998, miR-2941, miR-283 and miR-12, were downregulated in Groups B and D. Among the miRNAs that were upregulated, some, such as miR-622, miR-1767, miR-4448, miR-3809, miR-3888-5p and miR-2951, were also abundantly expressed before the ingestion of a blood meal, but the expression of others, such as miR-1951, miR-19c, miR-424, miR-103, miR-4728-5p, miR-193-5p, miR-976-5p and miR-3811e-5p, were expressed at low levels before the ingestion of a blood meal, and their expression increased rapidly after the ingestion of a blood meal.

**Fig. 4:**
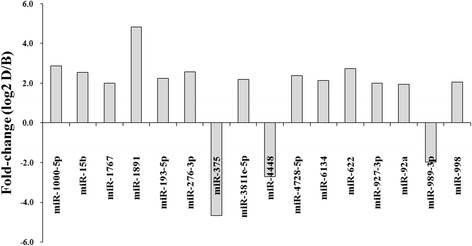
Relative expression of miRNAs with more than 2-fold difference in expression between the midguts of DENV-2-infected and uninfected mosquitoes. “Fold-change”is the magnitude of change in miRNA expression in the DENV-2-infected group relative to that in the uninfected group (fold-change = log_2_ratio)

By comparing miRNA expression levels in Group D to those in Group B, we identified 36 miRNAs that were upregulated and 2 that were downregulated in Group D; those with > 2- or < -2-fold changes in expression between these two groups are listed in Table 5. Aal-miR-1767, aal-miR-193-5p, aal-miR-276-3p, aal-miR-4728-5p, aal-miR-622 and aal-miR-4448 all showed significant differences in expression in Group D compared with their expression in Group B (Fig. 4), and the expression levels of these were high.

**Table 5:** Significantly modulated miRNAs that displayed a < -2- or > 2-fold difference in expression between the midguts of mosquitoes that had ingested a DENV-2-infected blood meal (D) and those that had ingested a regular blood meal (B)

miR-name	Reads (B)	Reads (D)	Normalized reads (B)	Normalized reads (D)	Fold-change	*P*-value
miR-1000-5p	20	159	1.39	10.09	2.9	0.00001
miR-15b	373	2404	25.94	152.52	2.6	0.00001
miR-1767	5149	21,657	358.14	1373.98	2.0	0.00001
miR-1891	1	31	0.07	1.97	4.8	0.00001
miR-193-5p	764	3934	53.14	249.58	2.2	0.00001
miR-276-3p	1394	9020	96.96	572.25	2.6	0.00001
miR-375	23	1	1.60	0.06	-4.7	0.00001
miR-3811e-5p	577	2895	40.13	183.67	2.2	0.00001
miR-4448	42,962	7183	2988.27	455.71	-2.7	0.00001
miR-4728-5p	444	2519	30.88	159.81	2.4	0.00001
miR-6134	777	3757	54.05	238.35	2.1	0.00001
miR-622	13,931	100,817	968.99	6396.11	2.7	0.00001
miR-927-3p	14	61	0.97	3.87	2.0	0.00001
miR-92a	183	776	12.73	49.23	2.0	0.00001
miR-989-3p	149	42	10.36	2.66	-2.0	0.00001
miR-998	205	936	14.26	59.38	2.1	0.00001

We chose the four miRNAs that displayed the greatest differences in expression between Groups B and D: aal-miR-1767 (GenBank KY062157), aal-miR-276-3p (GenBank KY062158), aal-miR-4448 andaal-miR-4728-5p (GenBank KY062160), for verification *via* stem-loop qRT-PCR.

### Verification of miRNAs *via* stem-loop qRT-PCR

qRT-PCR with a stem-loop primer confirmed the existence of all four of the previously mentioned miRNAs and verified that their expression levels differed significantly between the B and D treatment groups (Fig. 3d). In addition, the trends in their expression levels were consistent with those obtained *via* high-throughput deep sequencing. We randomly selected aal-miR-4728-5pfor further research.

### Enhanced DENV-2 replication in the C6/36 cell line by aal-miR-4728-5p

**Fig. 5:**
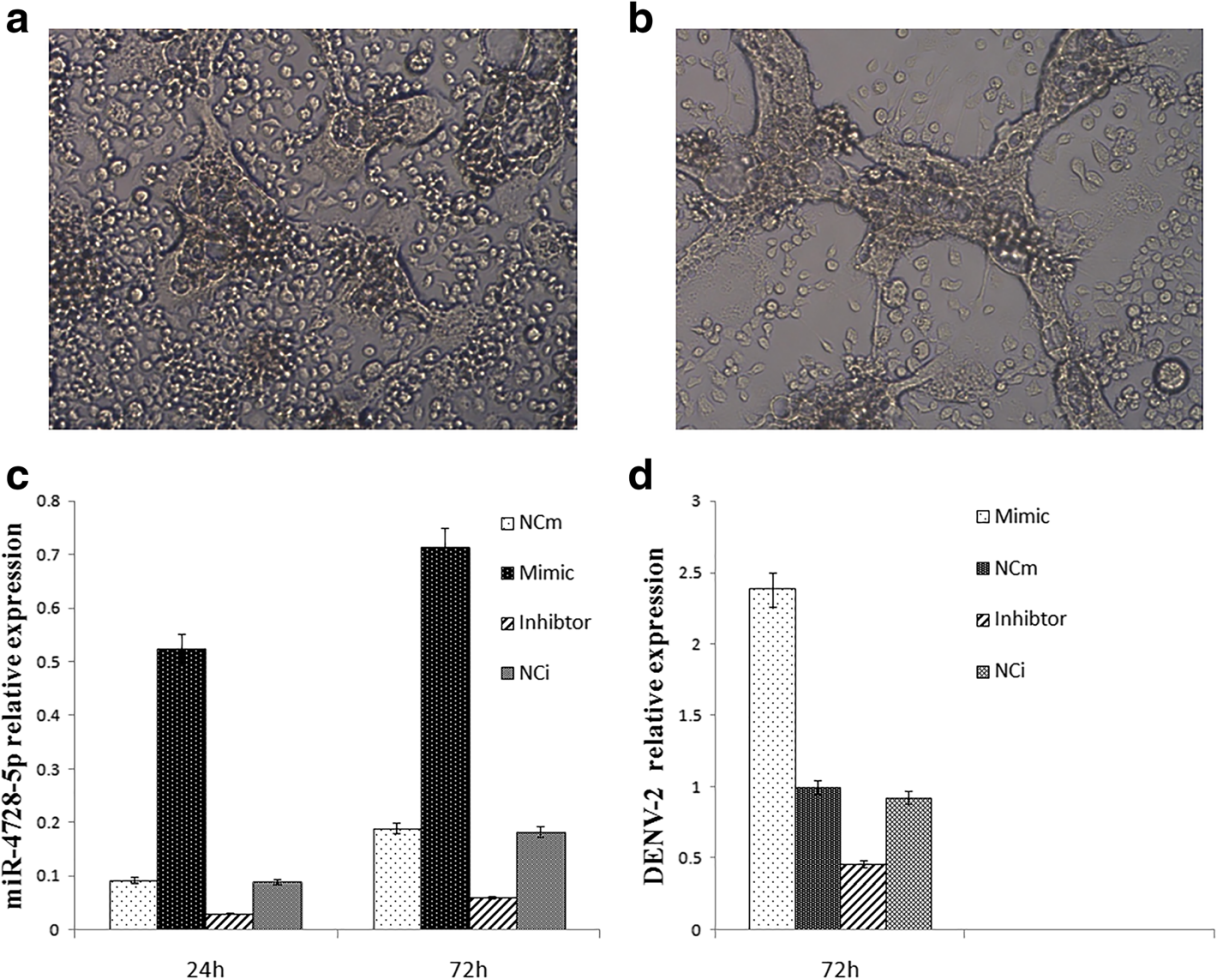
Effects of aal-miR-4728-5p on the replication of DENV-2 in C6/36 cells. **a** Cytopathic effect (CPE) of DENV-2 in C6/36 cells that had been transfected with an aal-miR-4728-5p inhibitor. **b** CPE in C6/36 cells that had been transfected with an aal-miR-4728-5p mimic. **c** Relative expression of aal-miR-4728-5p in C6/36 cells 24 h and 72 h post-transfection with the mimic, inhibitor and negative controls. **d** Relative DENV-2 expression levels in C6/36 cells after transfection with an aal-miR-4728-5p mimic or inhibitor. Relative expression levels of aal-miR-4728-5p and DENV-2 were determined *via* qRT-PCR and calculated using the 2^-⊿⊿CT^ method. *Abbreviations*: NCm, negative control for the aal-miR-4728-5p mimic; NCi, negative control for the aal-miR-4728-5p inhibitor

Because we had previously confirmed that aal-miR-4728-5p was also expressed in the *Ae. albopictus* C6/36 cell line (data not shown), we transfected C6/36 cells with an aal-miR-4728-5p synthetic mimic and inhibitor, as well as negative controls for the mimic (NCm) and inhibitor (NCi), and inoculated them with the DENV-2 virus 24 h post-transfection. The expression levels of aal-miR-4728-5p were measured 24 h and 72 h post-transfection, and the expression level of gene for the DENV-2 E protein gene was measured using DENV-2-specific primers (Table 1) 72 h post-inoculation. qRT-PCR results indicate that the expression of aal-miR-4728-5p increased by 3.09 and 2.41 times 24 h and 72 h, respectively, after the cells had been transfect with the aal-miR-4728-5p mimic, and its expression was decreased by 0.39 and 0.24 times at the same time points by its inhibitor (Fig. 5c). The relative expression of DENV-2 significantly increased in the mimic-transfected group (2.05 times; *t*_(2)_ = 6.406, *P* = 0.024) compared with its expression in the NCm group, and it decreased in the inhibitor-transfected group by 0.28 times (*t*_(2)_ = -7.727, *P* = 0.016) compared with its expression in the NCi group (Fig. 5d). Titres in the supernatant from the mimic groups were higher than those from the NCm group (*t*_(2)_ = 6.36, *P* = 0.024). Furthermore, the cytopathic effect (CPE) of DENV-2 on C6/36 cells was significantly greater in cells transfected with the aal-miR-4728-5p mimic than in those transfected with NCm 4 days post-inoculation (Fig. 5a, b). Collectively, these results show that aal-miR-4728-5p may play an important role in DENV infections in *Ae. albopictus*.

## Discussion

The present study provides the first verification of six miRNAs in *Ae. albopictus*: aal-miR-1174 (GenBank KY062161), aal-miR-2951 (GenBank KY062162), aal-miR-956, aal-miR-4728-5p (GenBank KY062160), aal-miR-1767 (GenBank KY062157) and aal-miR-4448 (GenBank KY062159). We did not detect the five previously reported *Ae. albopictus* miRNAs: aal-mir-286, aal-miR-315, aal-miR-309, aal-miR-929 and aal-mir-971. However, aal-mir-286 was only detected in embryos, aal-miR-315is highly expressed in embryos but not at other life-stages, and aal-miR-309, aal-miR-929 and aal-mir-971 are only weakly expressed in adult mosquitoes [37]. This suggests that these miRNAs are probably tissue and life-stage specific and are not expressed in the midgut of adult female *Ae. albopictus*. We found marked changes in miRNA expression following the ingestion of ablood meal compared with the changes in response to a sugar solution. MiRNAs upregulated following the ingestion of a blood meal (30/173; 17.3%) are related to the digestion of blood and the resistance to invasion by blood-borne pathogens. Interestingly, most miRNAs in the midguts of *Ae. albopictus*(96/173, 55.5%) were downregulated 24 h after the ingestion of a blood meal, including the majority of those that had been highly expressed. For example, the expression levels of miR-956-3p and miR-184in Group B were one order of magnitude lower than in Group C. This phenomenon has also been observed in other mosquito species; for example, the expression of miR-275 in the fat body of *Ae. aegypti* has been shown to first decline 24 h post-ingestion of a blood meal (pbm) and then increase 48 h pbm [38]. In our experiment, although miR-275 was downregulated 24 h pbm it was still expressed at a relatively high level. In contrast, the expression of miR-275 in the midgut of *Ae. aegypti* resulted in very low (reads < 10) and showed little increase 24 h pbm. With the exception of miR-275, we found that 7 miRNAs, miR-1175, miR-184, miR-281, miR-283, miR-317 and miR-34, showed the opposite trend, i.e. they were downregulated in the midgut of *Ae. albopictus* but were upregulated in the midgut of *Ae. aegypti* at the same time point pbm [39]. MiR-275 expression is upregulated in the body of *Ae. aegypti* pbm and has been found to be indispensable for blood digestion and egg development, but its function in *Ae. albopictus* is uncertain*.* We found that miR-375 was expressed in the midgut before the ingestion of a blood meal (15 reads), and its expression increased 24 h PBM (23 reads). In *Ae. aegypti*, miR-375 was not expressed in the midgut and was only detected in the bodies of blood-fed females. This difference between these two *Aedes* mosquitoes may partly reflect differences in the expression patterns and roles of different molecular factors.

Compared with expression in Group B, most of the miRNAs that were differentially expressed in Group D, such as miR-1175, miR-276, miR-317, miR-34-5p, miR-1767 and miR-375, were upregulated, although the expression of miR-989 decreased to a very low level. A recent study on infection of *Ae. aegypti* by DENV-2 indicates that among the 31 miRNAs with relatively marked differences in expression levels between Groups D and B, only four, miR-34-3p, miR-5119-5p, miR-87-5p, miR-988-5p, were upregulated; the remaining 27, including miR-1175, miR-276-5p, miR-281, miR-2945, miR-317 and miR-33-5p, were downregulated in Group D compared with their expression in Group C [21]. Another study found that the expression of miR-34, miR-1174 and miR-1175 in midgut epithelial cells of *An. gambiae* decreased, whereas that of miR-989 increased four-fold 24–48 h after *Plasmodium* infection [29]. These results indicate that some miRNAs, such as miR-1175, miR-34 and miR-989, may have different functions in different species; for example, miR-1175, miR-276 and miR-317 display contrary trends in their expression in *Ae. albopictus* and *Ae. aegypti*, and the corresponding molecular mechanisms may also be different in each species.

Among the miRNAs listed in Table 5, miR-375 enhances DENV infections in *Ae. aegypti* Aag2 cells, which suggests that it may be involved in DENV infection in this species [20]. In this study, the expression level of miR-375 in the midgut was too low to suggest that it plays a role in the process of DENV infection in the midgut of *Ae. albopictus*. Further more, miR-275 expression was upregulated in Group D by a ratio of 2.67 (1.37-fold) compared with its expression in Group B. This result, together with its function in *Ae. aegypti*, suggests that this miRNA may play a role in DENV infections in the midgut of *Ae. albopictus*.

We also found that many miRNAs that were expressed at very low levels, such as miR-252, showed significant differences in expression between Groups D and B. Although miR-252was upregulated in Group D, its expression levels were very low, suggesting that it is unlikely to play a role in midgut infections. In another study, miR-252 was found to be abundantly expressed in adult female *Ae. albopictus* and was downregulated 7 days after an intrapleural injection of DENV-2. Furthermore, a transient transfection assay showed that miR-252 inhibited the replication of DENV-2 in C6/36 cells [40]. These differences suggest that this miRNA may have different expression patterns in different organs in *Ae. albopictus*. However, it is also possible that the change in miRNA expression induced by oral infections could be different from those induced by intrapleural injections, which is not the natural pathway of infection.

Aal-miR-4728-5p, a conserved miRNA that was newly discovered in *Ae. albopictus*, was expressed in the midgut before the ingestion of a blood meal (109 reads), but its expression subsequently increased PBM in infected midguts (2519 reads) compared with its expression in uninfected midguts (444 reads). MiR-4728-5p is also expressed in humans, where it is involved in tumourigenesis [41]. There has been no previous research on the role of this miRNA in DENV infections, but our transient transfection experiments indicate that it enhances the replication of DENV-2 in C6/36 cells. We plan to conduct similar experiments on other miRNAs that displayed significant differences in expression between infected and uninfected mosquitoes. This work should improve our understanding of the miRNAs involved in the process of midgut infection by the DENV.

In summary, this study provides the first information on miRNAs in *Ae. albopictus* midgut and suggests potential avenues for further research on the role of these miRNAs during DENV infections in *Ae. albopictus*. In the absence of the complete *Ae. albopictus* genome, the majority of the miRNAs we found remain unidentified. Mapping our RNA libraries to the *Ae. albopictus* genome, when this becomes available, will help us determine the structure and function of all the reads obtained. A better understanding of the mechanisms responsible for the midgut infection barrier could lead to new insights in mosquito biology and novel approaches for combating mosquito-borne infectious diseases.

## Conclusion

The present study provides the first information on microRNAs expressed in the midgut of *Ae. albopictus* and describes species-specific changes in their expression levels following DENV-2 infection. It was confirmed that six miRNAs, aal-miR-1174, aal-miR-2951, aal-miR-956, aal-miR-4728-5p, aal-miR-1767 and aal-miR-4448, in the midguts of wild *Ae. albopictus* differ from those in laboratory strains. The aal-miR-4728-5p was chosen for an in vitro transient transfection assay, and the results show that this miRNA enhances DENV replication in C6/36 cells.

## Additional files

Additional file 1: Table S1. Conserved miRNAs and their expression in the midgut of *Ae. albopictus* mosquitoes that were fed with sugar, regular blood and DENV-2-infected blood. (DOCX 34 kb)
Additional file 2: Table S2. Name, sequence, location, normalized expression of miRNAs from the midguts adult female *Ae. albopictus* mosquitoes. (DOCX 34 kb)

## Abbreviations

aae-mir-1174: *Aedes aegypti* microRNA -1174 stem loop
C6/36: *Aedes albopictus* clone
cDNA: complementary deoxynucleic acid
Ct: Threshold cycle
DENV: Dengue virus
DENV-2: Dengue virus 2
DF: Dengue fever
DHF: Dengue haemorrhagic fever
DMEM: Dulbecco’s Modified Eagle’s Medium
dNTP: Deoxynucleotide triphosphate
FBS: Foetalbovine serum
miR-1174: microRNA-1174
miR-1175: microRNA-1175
miRNA: microRNA
miRNAs: microRNAs
PCR: Polymerase chain reaction
RT: Reverse transcription
sRNA: Small RNA
