# Supplementary material for: Identification of microRNAs expressed in the midgut of Aedes albopictus during dengue infection
Source: Parasit Vectors. 2017 Feb 3;10:63. doi: 10.1186/s13071-017-1966-2 (PMC5292000; doi:10.1186/s13071-017-1966-2)
Supplement: Additional file 1: Table S1. — Conserved miRNAs and their expression in the midgut of Ae. albopictus mosquitoes that were fed with sugar, regular blood and DENV-2-infected blood. (DOCX 34 kb) [file 13071_2017_1966_MOESM1_ESM.docx]

**Additional file 1: Table S1.**Conserved miRNAs and their expression in the midgut of *Ae. albopictus* mosquitoes that were fed with sugar, regular blood and DENV-2-infected blood

|  |  |  |  | **Reads^a^** |  |
| --- | --- | --- | --- | --- | --- |
| **Name** | **Sequence** | **Length** | **C** | **B** | **D** |
| aal-bantam-3p | TGAGATCATTTTGAAAGCTGATT | 23 | 3,867 | 491 | 1,190 |
| aal-bantam-5p | CCGGTTTTCATTTTCGATCTGACT | 24 | 50 | 12 | 25 |
| aal-let-7 | TGAGGTAGTTGGTTGTATAGT | 21 | 37,277 | 2,226 | 5,473 |
| aal-let-7-3p | TATGCAATCTGCTAGCTTGACG | 22 | 6 | 2 | 0 |
| aal-let-7b-5p | TGAGGTAGTAGGTTGTGTGGTT | 22 | 1,031 | 2,385 | 3,628 |
| aal-let-7f | TGAGGTAGTAGATTGTATAGT | 21 | 204 | 19,241 | 43,862 |
| aal-miR-1 | TGGAATGTAAAGAAGTATGTAG | 22 | 366 | 46 | 109 |
| aal-miR-1-3p | TGGAATGTAAAGAAGTATGGAG | 22 | 335,838 | 28,317 | 66,289 |
| aal-miR-1000-5p | ATATTGTCCTGTCACAGCAGTA | 22 | 16 | 20 | 159 |
| aal-miR-100-3p | CAAGAACGGATGTATGGGATTC | 22 | 1,722 | 359 | 688 |
| aal-miR-100-5p | AACCCGTAGATCCGAACTTGTG | 22 | 2,206 | 307 | 401 |
| aal-miR-103-3p | AGCAGCATTGTACAGGGCTATG | 22 | 19 | 2,646 | 4,954 |
| aal-miR-10-3p | CAAATTCGGTTCTAGAGAGGTTT | 23 | 339 | 12 | 58 |
| aal-miR-10-5p | ACCCTGTAGATCCGAATTTGTT | 22 | 66 | 3 | 11 |
| aal-miR-11-3p | CATCACAGTCTGAGTTCTTGC | 21 | 1,348 | 153 | 257 |
| aal-miR-11-5p | CAAGAACTCCGGCTGTGACCTGTG | 24 | 346 | 37 | 64 |
| aal-miR-1174 | TCAGATCTAACTAATACCCAACT | 23 | 5,080 | 815 | 2,672 |
| aal-miR-1174-3p | TGACGTCATGCTCGATTGGCTC | 22 | 11 | 6 | 5 |
| aal-miR-1175-3p | TGAGATTCTACTTCTCCGACT | 21 | 4,078 | 726 | 2,466 |
| aal-miR-1175-5p | AAGTGGAGTAGTGGTCTCATCG | 22 | 37,298 | 7,530 | 9,780 |
| aal-miR-122-5p | TGGAGTGTGACAATGGTGTTTG | 22 | 61 | 1 | 4 |
| aal-miR-12-3p | CAGTACTTATGTTATGCTCTCT | 22 | 19 | 1 | 5 |
| aal-miR-12-5p | TGAGTATTACATCAGGTACTGGT | 23 | 4,201 | 500 | 1,248 |
| aal-miR-124 | TAAGGCACGCGGTGAATGCCAA | 22 | 5 | 0 | 15 |
| aal-miR-125-3p | ACAAGTTTTGATCTCCGGTAT | 21 | 596 | 97 | 128 |
| aal-miR-125b-5p | TCCCTGAGACCCTAACTTGTGA | 22 | 1,066 | 159 | 191 |
| aal-miR-1260 | TATCCCACCGCTGCCACCA | 19 | 146 | 350 | 592 |
| aal-miR-1273f | GGAGTTGCGAGTTGCAGAG | 19 | 1,368 | 4,815 | 5,902 |
| aal-miR-133b | TTGGTCCCCTTCAACCAGCT | 20 | 10 | 2 | 9 |
| aal-miR-1357 | AGATTATGAGAGCTGAGGGCA | 21 | 337 | 1,541 | 1,794 |
| aal-miR-137-1-5p | ACGCGTATTCTTGGGTTATTAA | 22 | 9 | 1 | 13 |
| aal-miR-137-3p | TATTGCTTGAGAATACACGTAG | 22 | 125 | 15 | 35 |
| aal-miR-13a-3p | TATCACAGCCATTTTGATGAGCTC | 24 | 46 | 9 | 33 |
| aal-miR-13b-3p | TATCACAGCCATTTTGACGAGT | 22 | 915 | 216 | 598 |
| aal-miR-14 | TCAGTCTTTTTCTCTCTCCTA | 21 | 1,952 | 387 | 591 |
| aal-miR-1420b-5p | TGTCCAATCTGGGAGCACTCC | 21 | 90 | 597 | 2,019 |
| aal-miR-1421al-5p | CAGGATCATTGGTACAGCAA | 20 | 5,784 | 1,506 | 2,562 |
| aal-miR-15-3p | CTGGTTTCTGTTGGTCTT | 18 | 7,893 | 3,879 | 4,741 |
| aal-miR-15b | TAGCAGCACATCATGGTTTA | 20 | 14 | 373 | 2,404 |
| aal-miR-1587 | TGGGCTGCGCATGGGTGGGG | 20 | 20,568 | 3,490 | 7,420 |
| aal-miR-1614-3p | AAGGGAGGAACATGAAGCAGA | 21 | 13,938 | 3,567 | 4,128 |
| aal-miR-1767 | AGACAGGAGAACAGCAAGGT | 20 | 1,660 | 5,149 | 21,657 |
| aal-miR-184-3p | TGGACGGAGAACTGATAAGGGC | 22 | 720,196 | 52,359 | 91,873 |
| aal-miR-184-5p | CCTTATCATTCTTTCGCCCCGT | 22 | 16 | 9 | 6 |
| aal-miR-1889-3p | CACGTTACAGATTGGGGTTTCC | 22 | 53 | 5 | 8 |
| aal-miR-1889-5p | TAATCTCAAATTGTAACAGTGG | 22 | 125 | 23 | 64 |
| aal-miR-1890 | TGAAATCTTTGATTAGGTCTGG | 22 | 23 | 5 | 5 |
| aal-miR-1891 | TGAGGAGTTAATTTGCGTGTTT | 22 | 14 | 1 | 31 |
| aal-miR-190-3p | CCCAGGAATCAAACATATTATTA | 23 | 27 | 6 | 10 |
| aal-miR-190-5p | AGATATGTTTGATATTCTTGGTTG | 24 | 1,870 | 160 | 310 |
| aal-miR-193 | AACTGGCCTACAAAGTCCCAG | 21 | 2 | 13 | 0 |
| aal-miR-193-5p | TGGGCTTGCGGGCGACTTG | 19 | 61 | 764 | 3,934 |
| aal-miR-1951 | GATAGTAAGACTGCTGTGGCTA | 22 | 208 | 2,388 | 6,421 |
| aal-miR-1957a | CAGTGGTAGAGCATTCGACT | 20 | 616 | 1,384 | 1,976 |
| aal-miR-19c | TGGCACTCAATGCAAAACTCG | 21 | 48 | 2,480 | 4,841 |
| aal-miR-210 | CTGTGCGTGTGACAGCGGCTGA | 22 | 0 | 23 | 31 |
| aal-miR-2356 | TCTTGGTGAGGGAGTCTGGAG | 21 | 12,982 | 4,502 | 5,200 |
| aal-miR-241-5p | GGAGGTAGTGACGAGAAATAA | 21 | 5,632 | 55,258 | 53,572 |
| aal-miR-2423 | TTGGTCGGTTGTTGTTTTCAT | 21 | 4,811 | 408 | 1,055 |
| aal-miR-2449 | TGGGCAGGAGCTAGTCAGGTGC | 22 | 18,221 | 6,194 | 8,189 |
| aal-miR-252-5p | CTAAGTACTAGTGCCGCAGGAG | 22 | 42 | 9 | 21 |
| aal-miR-252 | CGAAGTAGTATGCGTAGATGA | 21 | 21 | 2 | 8 |
| aal-miR-25-3p | CATTGCACTTGTCTCGGTCTGA | 22 | 0 | 1,488 | 2,929 |
| aal-miR-25-5p | AGGCGGAGACTTGGGCAATTGCT | 23 | 4 | 31 | 53 |
| aal-miR-263a-3p | CGTGTTCTGGCAGTGGCATCCC | 22 | 11 | 1 | 7 |
| aal-miR-263a-5p | AATGGCACTGGAAGAATTCACGG | 23 | 144 | 30 | 44 |
| aal-miR-275-3p | TCAGGTACCTGAAGTAGCGCGCG | 23 | 84,533 | 22,782 | 60,766 |
| aal-miR-275-5p | CGCGCTAAGCAGGAACCGAGACT | 23 | 197 | 75 | 102 |
| aal-miR-276-3p | TAGGAACTTCATACCGTGCTC | 21 | 4,854 | 1,394 | 9,020 |
| aal-miR-276-5p | AGCGAGGTATAGAGTTCCTACG | 22 | 5,389 | 638 | 1,805 |
| aal-miR-2765 | TGGTAACTCCACCACCGTTGGC | 22 | 129 | 38 | 73 |
| aal-miR-277-3p | TAAATGCACTATCTGGTACGACA | 23 | 743 | 113 | 298 |
| aal-miR-277-5p | CGTGTCAGAAGTGCATTTACA | 21 | 27 | 1 | 13 |
| aal-miR-2779 | CATCCGGCTCGAAGGACCA | 19 | 666 | 2,464 | 3,669 |
| aal-miR-278-3p | TCGGTGGGACTTTCGTCCGTTT | 22 | 22 | 5 | 3 |
| aal-miR-278-5p | ACGGACGATAGTCTTCAGCGGCC | 23 | 34 | 16 | 42 |
| aal-miR-279 | TGACTAGATCCACACTCATCCA | 22 | 1 | 0 | 1 |
| aal-miR-279-3p | TGACTAGATCCACACTCATTAA | 22 | 673 | 86 | 277 |
| aal-miR-2796-3p | GTAGGCCGGCGGAAACTACTTG | 22 | 15,877 | 817 | 2,819 |
| aal-miR-2796-5p | AGGGGTTTCTTTCGGCCTCCAG | 22 | 8 | 4 | 4 |
| aal-miR-281-2-5p | AAGAGAGCTATCCGTCGACAGT | 22 | 249,836 | 23,630 | 46,766 |
| aal-miR-281-3p | CTGTCATGGAATTGCTCTCTT | 21 | 2,079 | 311 | 498 |
| aal-miR-283 | CAATATCAGCTGGTAATTCTG | 21 | 877 | 111 | 198 |
| aal-miR-283-5p | AATATCAGCTGGTAATTCTG | 20 | 949 | 113 | 198 |
| aal-miR-285 | TAGCACCATTCGAAATCAGTAC | 22 | 2 | 3 | 4 |
| aal-miR-2940-3p | TGTCGACAGGGAGATAAATCACT | 23 | 3,867 | 1,452 | 3,114 |
| aal-miR-2940-5p | TGGTTTATCTTATCTGTCGAGGCA | 24 | 84,587 | 57,746 | 90,573 |
| aal-miR-2941 | TAGTACGGCTAGAACTCCACGGA | 23 | 380 | 31 | 20 |
| aal-miR-2942 | TATTCGAGACTTCACGAGTTAAT | 23 | 319 | 69 | 105 |
| aal-miR-2943 | TTAAGTAGGCACTTGCAGGCAA | 22 | 215 | 20 | 38 |
| aal-miR-2944 | TATCACAGCAGTAGTTACCTGGTA | 24 | 0 | 0 | 1 |
| aal-miR-2944b-5p | GAAGGAACTCCCGGTGTGATAT | 22 | 28 | 2 | 4 |
| aal-miR-2945-3p | TGACTAGAGGCAGACTCGTTT | 21 | 24,770 | 2,399 | 4,997 |
| aal-miR-2945-5p | AGCGGGTCTGTTTCTAGTGTCATG | 24 | 14 | 2 | 7 |
| aal-miR-2946 | TAGTACGGAAAAGATATGGGGA | 22 | 17 | 1 | 1 |
| aal-miR-2951-5p | AAGAGCTCAGCACGCAGGGGCGA | 23 | 14,468 | 96,318 | 70,082 |
| aal-miR-2-5p | CACAAGCGGTGGATGATGTG | 20 | 375 | 352 | 760 |
| aal-miR-2a-3p | TATCACAGCCAGCTTTGAAGA | 21 | 1,188 | 242 | 482 |
| aal-miR-2a-5p | ACTCTCAAAGTGGCTGTGAAAT | 22 | 276 | 58 | 73 |
| aal-miR-2b | TCACAGCCAGCTTTGATGAGC | 21 | 1,095 | 636 | 1,534 |
| aal-miR-2c | TATCACAGCCAGCTTTGAAG | 20 | 1,162 | 228 | 471 |
| aal-miR-305-3p | CGGCACATGTTGGAGTACACTTA | 23 | 1,288 | 671 | 869 |
| aal-miR-305-5p | ATTGTACTTCATCAGGTGCTCTGG | 24 | 639 | 380 | 781 |
| aal-miR-306-3p | GAGAGCACCTCGGTATCTAAGC | 22 | 12 | 2 | 6 |
| aal-miR-306-5p | TCAGGTACTGAGTGACTCTCA | 21 | 100 | 13 | 48 |
| aal-miR-308 | AATCACAGGAGTATACTGTGAG | 22 | 39 | 5 | 10 |
| aal-miR-308-5p | CGCGGTATATTCTTGTGGCTTG | 22 | 21,212 | 4,665 | 7,134 |
| aal-miR-3100-5p | TTGGGAACGGGATGGCTTGGG | 21 | 74,098 | 8,702 | 14,490 |
| aal-miR-31-3p | AGCTATTCAACTTCTTGTCTAT | 22 | 133 | 17 | 54 |
| aal-miR-31-5p | TGGCAAGATGTTGGCATAGCTG | 22 | 11,691 | 1,235 | 2,532 |
| aal-miR-316 | TGTCTTTTTCCGCTTACTGCCG | 22 | 822 | 223 | 722 |
| aal-miR-316-5p | TGTCTTTTTCCGCTTACTGC | 20 | 846 | 229 | 722 |
| aal-miR-317 | TGAACACAGCTGGTGGTATCTCA | 23 | 111,302 | 7,866 | 19,033 |
| aal-miR-317-3p | TGAACACAGCTGGTGGTATCTCAG | 24 | 93,736 | 6,753 | 16,402 |
| aal-miR-33a-5p | GTGCATTGTAGTTGCATTGCA | 21 | 3,548 | 401 | 1,058 |
| aal-miR-34-3p | CAACCACTATCCGCCCTGCCGCC | 23 | 117 | 15 | 44 |
| aal-miR-34-5p | TGGCAGTGTGGTTAGCTGGTT | 21 | 263,407 | 10,384 | 29,278 |
| aal-miR-375 | TTTGTTCGTTCGGCTCGCGTGA | 22 | 15 | 23 | 1 |
| aal-miR-3809-3p | GTAGGTCAGTTGGTTGGA | 18 | 29 | 28 | 39 |
| aal-miR-3809-5p | TCAGAACTTGCACGGCTGA | 19 | 6,010 | 41,863 | 49,671 |
| aal-miR-3811e-5p | TTGGCTTCCTTGCGGTGCACA | 21 | 72 | 577 | 2,895 |
| aal-miR-3870-5p | TGGAGACACAAGAAGGAGAAA | 21 | 12,083 | 3,165 | 4,533 |
| miR-4110-5p | AACCAGAGAATAGCAGTGTG | 20 | **12** | 304 | 1,126 |
| aal-miR-4175-3p | GGGATGTAGCTCAGATGGTAG | 21 | 54,667 | 154,660 | 140,725 |
| aal-miR-424-3p | CAAGAAGATGAGGCCTGCTAT | 21 | **102** | 1,140 | 4,029 |
| aal-miR-4443 | TTTGAGGGCGTGGGTTTT | 18 | 1,172 | 730 | 818 |
| aal-miR-4448 | GGCTCGTTGGTCTAGGGGT | 19 | 4,459 | 42,962 | 7,183 |
| aal-miR-4728-5p | TGGGAGGGCAGAGGGGCAGCA | 21 | 109 | 444 | 2,519 |
| aal-miR-493-3p | TCAAGTCTAACTGTGTGGCAG | 21 | 39,546 | 14,154 | 19,572 |
| aal-miR-493-5p | TTGCCATGGATGCTTTCATT | 20 | 12 | 180 | 415 |
| aal-miR-5706 | TCTGGATACAATGCTGAACT | 20 | 75,385 | 34,983 | 47,242 |
| aal-miR-6086 | GGAGGTTGGGTCGGGCCAGG | 20 | 12,627 | 3,640 | 5,052 |
| aal-miR-622 | AGAGTTCACTGGGTTGGAGGC | 21 | 15,010 | 13,931 | 100,817 |
| aal-miR-6666-3p | TCGGGTGATGCGGTAGAGGA | 20 | **773** | 56 | 154 |
| aal-miR-6668-3p | TGGGATGTGGAATAGATTGGG | 21 | 36,342 | 6,488 | 8,880 |
| aal-miR-7 | TGGAAGACTAGTGATTTTGTTGTT | 24 | 99 | 40 | 52 |
| aal-miR-71-3p | TCTCACTACCTTGTCTTTCATG | 22 | 46 | 18 | 34 |
| aal-miR-71-5p | AGAAAGACATGGGTAGTGAGATA | 23 | 11,330 | 877 | 1,986 |
| aal-miR-79 | TAAAGCTAGATTACCAAAGCA | 21 | 2,565 | 784 | 1,395 |
| aal-miR-79-3p | TAAAGCTAGATTACCAAAGCAT | 22 | 2,534 | 773 | 1,380 |
| aal-miR-8-3p | TAATACTGTCAGGTAAAGATGTC | 23 | 25,074 | 5,988 | 9,934 |
| aal-miR-8-5p | CATCTTACCGGGCAGCATTAGA | 22 | 10,856 | 656 | 1,491 |
| aal-miR-9 | TCTTTGGTTATCTAGCTGTAT | 21 | 941 | 32 | 79 |
| aal-miR-927-3p | TAAGGCTTTGGATTGCTAA | 19 | 31 | 14 | 61 |
| aal-miR-927-5p | TTTAGAATTCCTACGCTTTACC | 22 | 8 | 2 | 1 |
| aal-miR-92a | TATTGCACTTGTCCCGGCCTAT | 22 | 537 | 183 | 776 |
| aal-miR-92a-5p | CGGTACGGACAGGGGCAATATT | 22 | 478 | 81 | 74 |
| aal-miR-92b | AATTGCACTTGTCCCGGCCTG | 21 | 360 | 54 | 133 |
| aal-miR-932-3p | TGCAAGCAATGTGGAAGTGAAG | 22 | 127 | 4 | 15 |
| aal-miR-956-3p | TTTCGAGACCACTGCAAATCATT | 23 | 1,793,142 | 79,819 | 164,004 |
| aal-miR-956-5p | GTTTGAAATGGTCTCGTTAACT | 22 | 286 | 56 | 56 |
| aal-miR-965 | TAAGCGTATAGCTTTTCCCATT | 22 | 53 | 11 | 61 |
| aal-miR-970-3p | TCATAAGACACACGCGGCTAT | 21 | 22,112 | 1,615 | 3,709 |
| aal-miR-976-5p | CTCTATGCAGTGCGCGCGGCT | 21 | 267 | 5,769 | 13,635 |
| aal-miR-980-3p | TAGCTGCCTAGTGAAGGGCAAT | 22 | 7 | 0 | 1 |
| aal-miR-980-5p | GGGTTCGTGACTGGGGTAG | 19 | 48 | 114 | 256 |
| aal-miR-988-3p | CCCCTTGTTGCAAACCTCACGC | 22 | 201 | 46 | 93 |
| aal-miR-989 | TGTGATGTGACGTAGTGGTAC | 21 | 2,897 | 133 | 42 |
| aal-miR-993 | TACCCTGTAGTTCCGGGCTTTT | 22 | 5 | 0 | 2 |
| aal-miR-993-3p | GAAGCTCGTCTCTACAGGTAT | 21 | 7 | 3 | 6 |
| aal-miR-996 | TGACTAGATTACATGCTCGTCT | 22 | 6,386 | 589 | 1,214 |
| aal-miR-998 | TAGCACCATGAGATTCAGC | 19 | 1,493 | 205 | 936 |
| aal-miR-998-5p | ACTGAACTCTCGTGGGTCTGCA | 22 | 2,583 | 445 | 777 |
| aal-miR-999-3p | TGTTAACTGTAAGACTGTGTCT | 22 | 345 | 26 | 71 |
| aal-miR-9a-3p | TAAAGCTAGCATACCGAAGTTA | 22 | 84 | 13 | 33 |
| aal-miR-9a-5p | TCTTTGGTTATCTAGCTGTATGA | 23 | 908 | 31 | 73 |
| aal-miR-9b-3p | ATAAAGCTAGATTACCAAAGCA | 22 | 2,502 | 750 | 1,350 |
| aal-miR-9b-5p | TCTTTGGTGATTTTAGCTGTATG | 23 | 211 | 27 | 52 |
| aal-miR-9c-3p | TAAAGCTTTAGTACCAGAGGTC | 22 | 752 | 72 | 178 |
| aal-miR-9c-5p | TCTTTGGTATTCTAGCTGTAGA | 22 | 2,190 | 159 | 381 |
| aal-miR-9d-3p | ATAAAGCTAGATTACCAAAG | 20 | 2,504 | 749 | 1,356 |
| aal-miR-iab-4-3p | CGGTATACCTTCAGTATACGTA | 22 | 9 | 1 | 6 |
| aal-miR-iab-4-5p | ACGTATACTGAATGTATCCTGA | 22 | 100 | 13 | 38 |

^a^Reads by high throughput deep sequencing

C: Midguts from mosquitoes ingested with sugar solution

B: Midguts from mosquitoes ingested with regular blood meal

D: Midguts from mosquitoes ingested with artificial DENV-2 blood meal
