# Supplementary material for: Identification of microRNAs expressed in the midgut of Aedes albopictus during dengue infection
Source: Parasit Vectors. 2017 Feb 3;10:63. doi: 10.1186/s13071-017-1966-2 (PMC5292000; doi:10.1186/s13071-017-1966-2)
Supplement: Additional file 2: Table S2. — Name, sequence, location, normalized expression of miRNAs from the midguts adult female Ae. albopictus mosquitoes. (DOCX 34 kb) [file 13071_2017_1966_MOESM2_ESM.docx]

**Additional file 2: Table S2.** Name, sequence, location, normalized expression of miRNAs from the midguts adult female *Ae.albopictus* mosquitoes

| **miRNA family** | **Sequence** | **Supercontig** | **Normalized reads^a^** | | |
| --- | --- | --- | --- | --- | --- |
|  |  |  | **C1** | **B1** | **D1** |
| aal-bantam-3p | TGAGATCATTTTGAAAGCTGATT | 1.490 | 193.37 | 24.55 | 59.51 |
| aal-bantam-5p | CCGGTTTTCATTTTCGATCTGACT | 1.490 | 2.57 | 0.83 | 1.59 |
| aal-let-7 | TGAGGTAGTTGGTTGTATAGT | 1.430 | 1,917.07 | 154.83 | 347.22 |
| aal-miR-1-3p | TGGAATGTAAAGAAGTATGGAG | 1.812 | 17,271.37 | 1,969.62 | 4,205.56 |
| aal-miR-10-3p | CAAATTCGGTTCTAGAGAGGTTT | 1.440 | 17.43 | 0.83 | 3.68 |
| aal-miR-10-5P | ACCCTGTAGATCCGAATTTGTT | 1.440 | 3.39 | 0.35 | 0.70 |
| aal-miR-100 | AACCCGTAGATCCGAACTTGTG | 1.430 | 114.84 | 21.42 | 26.65 |
| aal-miR-100-3p | CAAGAACGGATGTATGGGATTC | 1.430 | 88.56 | 24.97 | 43.65 |
| aal-miR-1000-5p | ATATTGTCCTGTCACAGCAGTA | 1.187 | 0.82 | 1.39 | 10.09 |
| aal-miR-11 | CATCACAGTCTGAGTTCTTGCT | 1.744 | 70.66 | 10.78 | 17.00 |
| aal-miR-11-5p | CAAGAACTCCGGCTGTGACCTGTG | 1.744 | 17.79 | 2.57 | 4.06 |
| aal-miR-1175 | TGAGATTCTACTTCTCCGACTTAA | 1.125 | 202.06 | 46.95 | 142.62 |
| aal-miR-1175-5p | AAGTGGAGTAGTGGTCTCATCG | 1.125 | 1,918.15 | 523.76 | 620.47 |
| aal-miR-12-3p | CAGTACTTATGTTATGCTCTCT | 1.680 | 61.71 | 8.49 | 20.87 |
| aal-miR-12-5p | TGAGTATTACATCAGGTACTGGT | 1.680 | 216.05 | 34.78 | 79.18 |
| aal-miR-124 | TAAGGCACGCGGTGAATGCCAA | 1.600 | 0.01 | 2.30 | 0.01 |
| aal-miR-125-3p | ACAAGTTTTGATCTCCGGTAT | 1.430 | 30.65 | 6.75 | 8.12 |
| aal-miR-125-5p | TCCCTGAGACCCTAACTTGTGA | 1.430 | 54.82 | 11.06 | 12.12 |
| aal-miR-13-3p | TATCACAGCCATTTTGACGAGTT | 1.268 | 47.42 | 15.16 | 38.26 |
| aal-miR-13-5p | TCGTAAAAATGGTTGTGCTGTG | 1.268 | 18.62 | 4.73 | 7.80 |
| aal-miR-133b | TTGGTCCCCTTCAACCAGCT | 1.778 | 0.00 | 0.02 | 0.02 |
| aal-miR-137-1-3p | TATTGCTTGAGAATACACGTAG | 1.119 | 6.43 | 1.04 | 2.22 |
| aal-miR-137-1-5p | ACGCGTATTCTTGGGTTATTAAC | 1.119 | 6.53 | 1.04 | 6.53 |
| aal-miR-14 | TCAGTCTTTTTCTCTCTCCTA(T) | 1.249 | 100.39 | 26.92 | 37.49 |
| aal-miR-184 | TGGACGGAGAACTGATAAGGGC | 1.496 | 38,078.96 | 3,730.23 | 5,988.05 |
| aal-miR-1889-3p | CACGTTACAGATTGGGGTTTCC | 1.680 | 2.73 | 0.35 | 0.50 |
| aal-miR-1889-5p | TAATCTCAAATTGTAACAGTGG | 1.680 | 6.43 | 1.60 | 4.06 |
| aal-miR-1890 | TGAAATCTTTGATTAGGTCTGG | 1.204 | 1.18 | 0.35 | 0.35 |
| aal-miR-1891 | TGAGGAGTTAATTTGCGTGTTT | 1.466 | 0.91 | 0.00 | 1.97 |
| aal-miR-190 | AGATATGTTTGATATTCTTGGTTGT | 1.195 | 96.22 | 11.41 | 20.37 |
| aal-miR-190-3p | CCCAGGAATCAAACATATTATTA | 1.195 | 1.39 | 0.42 | 0.76 |
| aal-miR-193 | AACTGGCCTACAAAGTCCCAG | 1.436 | 0.00 | 0.00 | 0.00 |
| aal-miR-193-5p | TGGGCTTGCGGGCGACTTG | 1.436 | 3.14 | 53.14 | 249.58 |
| aal-miR-210-3p | TTGTGCGTGTGACAACGGCTAT | 1.512 | 0.01 | 1.60 | 1.97 |
| aal-miR-252-5p | CTAAGTACTAGTGCCGCAGGAG | 1.560 | 2.16 | 0.63 | 1.33 |
| aal-miR-252-3p | CTGCTGCCCAAGTGCTTATCG | 1.560 | 0.01 | 0.00 | 0.00 |
| aal-miR-263a-3p | CGTGTTCTGGCAGTGGCATCCC | 1.981 | 7.41 | 2.09 | 2.79 |
| aal-miR-263a-5p | AATGGCACTGGAAGAATTCACGG | 1.981 | 7.41 | 2.09 | 2.79 |
| aal-miR-275-3p | TCAGGTACCTGAAGTAGCGCG | 1.240 | 4,733.40 | 1,718.11 | 4,130.06 |
| aal-miR-275-5p | CGCGCTAAGCAGGAACCGAGACT | 1.240 | 10.13 | 5.22 | 6.47 |
| aal-miR-276-3p | TAGGAACTTCATACCGTGCTCT | 1.134 | 129.63 | 96.96 | 572.25 |
| aal-miR-276-5p | AGCGAGGTATAGAGTTCCTACG | 1.134 | 277.14 | 44.38 | 114.51 |
| aal-miR-2765 | TGGTAACTCCACCACCGTTGGC | 1.100 | 6.63 | 2.64 | 4.63 |
| aal-miR-277-3p | TAAATGCACTATCTGGTACGACA | 1.265 | 38.21 | 7.86 | 18.91 |
| aal-miR-277-5p | CGTGTCAGAAGTGCATTTACA | 1.265 | 1.39 | 0.07 | 0.62 |
| aal-miR-278-3p | TCGGTGGGACTTTCGTCCGTTT | 1.160 | 1.13 | 0.35 | 0.14 |
| aal-miR-278-5p | ACGGACGATAGTCTTCAGCGGCC | 1.160 | 1.02 | 1.98 | 2.66 |
| aal-miR-279-5p | GATGGGTGTGAATCTAGTGTTTCACA | 1.437 | 0.05 | 1.65 | 2.14 |
| aal-miR-279-3p | TGACTAGATCCACACTCATTAA | 1.437 | 34.61 | 5.98 | 17.57 |
| aal-miR-2796-5p | AGGGGTTTCTTTCGGCCTCCAG | 1.309 | 0.97 | 0.53 | 0.46 |
| aal-miR-2796-3p | GTAGGCCGGCGGAAACTACTTG | 1.309 | 834.26 | 58.50 | 185.13 |
| aal-miR-281-3p | TGTCATGGAATTGCTCTCTTTA | 1.957 | 106.92 | 21.63 | 31.59 |
| aal-miR-281-5p | AAGAGAGCTATCCGTCGACAGT | 1.957 | 12,820.51 | 1,636.80 | 2,943.81 |
| aal-miR-283 | CAATATCAGCTGGTAATTCTG | 1.680 | 45.10 | 7.72 | 12.56 |
| aal-miR-283-5p | AATATCAGCTGGTAATTCTG | 1.680 | 48.80 | 7.86 | 12.56 |
| aal-miR-285 | TAGCACCATTCGAAATCAGTAC | 1.230 | 0.00 | 0.00 | 0.00 |
| aal-miR-2940-3p | TGTCGACAGGGAGATAAATCACT | 1.222 | 198.87 | 101.00 | 197.56 |
| aal-miR-2940-5p | TGGTTTATCTTATCTGTCGAGGCA | 1.222 | 4,350.11 | 4,016.59 | 5,746.21 |
| aal-miR-2941 | TAGTACGGCTAGAACTCCACGGA | 1.385 | 19.54 | 2.16 | 1.27 |
| aal-miR-2942 | TATTCGAGACTTCACGAGTTAAT | 1.245 | 16.41 | 4.80 | 6.66 |
| aal-miR-2943 | TTAAGTAGGCACTTGCAGGCAA | 1.348 | 11.06 | 1.39 | 2.41 |
| aal-miR-2944b-5p | GAAGGAACTCCCGGTGTGATAT | 1.464 | 1.44 | 0.14 | 2.88 |
| aal-miR-2945-3p | TGACTAGAGGCAGACTCGTTT | 1.430 | 1,273.86 | 166.87 | 317.02 |
| aal-miR-2945-5p | AGCGGGTCTGTTTCTAGTGTCATG | 1.430 | 0.15 | 0.89 | 0.44 |
| aal-miR-2946 | TAGTACGGAAAAGATATGGGGA | 1.385 | 0.00 | 0.20 | 0.00 |
| aal-miR-2a | TCACAGCCAGCTTTGATGAGC（A） | 1.268 | 99.56 | 47.30 | 99.48 |
| aal-miR-2a-3p | TATCACAGCCAGCTTTGAAGA | 1.268 | 61.10 | 16.83 | 30.58 |
| aal-miR-2a-5p | ACTCTCAAAGTGGCTGTGAAAT | 1.268 | 14.19 | 4.03 | 4.63 |
| aal-mir-2b | TATCACAGCCAGCTTTGATGAGCT | 1.268 | 56.31 | 44.24 | 97.32 |
| aal-miR-305-3p | CGGCACATGTTGGAGTACACTTA | 1.240 | 66.24 | 46.67 | 55.13 |
| aal-miR-305-5p | ATTGTACTTCATCAGGTGCTCTGG | 1.240 | 32.86 | 26.43 | 49.55 |
| aal-miR-306-3p | GAGAGCACCTCGGTATCTAAGC | 1.785 | 0.00 | 0.14 | 0.00 |
| aal-miR-306-5p | TCAGGTACTGAGTGACTCTCA | 1.785 | 5.14 | 0.90 | 3.05 |
| aal-miR-308 | AATCACAGGAGTATACTGTGAG | 1.107 | 2.01 | 0.35 | 4.01 |
| aal-miR-308-5p | CGCGGTATATTCTTGTGGCTTG | 1.107 | 1,090.88 | 324.48 | 452.60 |
| aal-miR-31 | TGGCAAGATGTTGGCATAGCTGA | 1.636 | 590.80 | 82.22 | 155.75 |
| aal-miR-31-3p | AGCTATTCAACTTCTTGTCTAT | 1.636 | 6.84 | 1.18 | 3.43 |
| aal-miR-316 | TGTCTTTTTCCGCTTACTGCCG | 1.289 | 42.27 | 15.51 | 45.81 |
| aal-miR-317-3p | TGAACACAGCTGGTGGTATCTCA(G) | 1.265 | 4,820.63 | 469.71 | 1,040.59 |
| aal-miR-317-5p | GGGATACACCCTGTGCTCGCT | 1.265 | 3.75 | 0.42 | 0.82 |
| aal-miR-33 | GTGCATTGTAGTTGCATTGCA | 1.487 | 186.17 | 28.52 | 68.96 |
| aal-miR-34-3p | CAACCACTATCCGCCCTGCCGCC | 1.265 | 6.02 | 1.04 | 2.79 |
| aal-miR-34-5p | TGGCAGTGTGGTTAGCTGGTT | 1.265 | 13,546.41 | 722.27 | 1,857.48 |
| aal-miR-375 | TTTGTTCGTTCGGCTCGCGTGA | 1.435 | 0.77 | 1.60 | 0.06 |
| aal-miR-7 | TGGAAGACTAGTGATTTTGTTGTT | 1.136 | 5.09 | 2.78 | 3.30 |
| aal-miR-71 | AGAAAGACATGGGTAGTGAGATA(T) | 1.268 | 582.68 | 61.00 | 126.00 |
| aal-miR-71-3p | TCTCACTACCTTGTCTTTCATG | 1.268 | 2.37 | 1.25 | 2.16 |
| aal-miR-79-3p | TAAAGCTAGATTACCAAAGCAT | 1.785 | 130.32 | 53.77 | 87.55 |
| aal-miR-8-3p | TAATACTGTCAGGTAAAGATGTC | 1.411 | 1,289.50 | 416.50 | 630.24 |
| aal-miR-8-5p | CATCTTACCGGGCAGCATTAGA | 1.411 | 558.30 | 45.63 | 94.59 |
| aal-miR-87 | GTGAGCAAATTTTCAGGTGTGT | 1.360 | 1.70 | 0.83 | 2.22 |
| aal-miR-927-5p | TTTAGAATTCCTACGCTTTACC | 1.260 | 1.59 | 0.97 | 3.87 |
| aal-miR-92a | TATTGCACTTGTCCCGGCCTA(T) | 1.116 | 27.15 | 9.81 | 37.11 |
| aal-miR-92a-5p | CGGTACGGACAGGGGCAATATT | 1.116 | 24.58 | 5.63 | 4.69 |
| aal-miR-92b-3p | AATTGCACTTGTCCCGGCCTG | 1.116 | 18.51 | 3.76 | 8.44 |
| aal-miR-932-5p | TCAATTCCGTAGTGCATTGCAG | 1.1064 | 0.51 | 0.00 | 0.00 |
| aal-miR-932-3p | TGCAAGCAATGTGGAAGTGAAG | 1.106 | 6.53 | 0.28 | 1.21 |
| aal-miR-965 | TAAGCGTATAGCTTTTCCCATT | 1.510 | 2.73 | 0.77 | 3.87 |
| aal-miR-970-3p | TCATAAGACACACGCGGCTAT | 1.229 | 1,137.17 | 112.33 | 235.31 |
| aal-miR-980-3p | TAGCTGCCTAGTGAAGGGCAAT | 1.230 | 2.47 | 7.93 | 16.24 |
| aal-miR-988-5p | GTGTGCTTTGTGACAATGAGAT | 1.442 | 0.31 | 0.00 | 0.00 |
| aal-miR-988-3p | CCCCTTGTTGCAAACCTCACGC | 1.442 | 10.34 | 3.20 | 5.90 |
| aal-miR-989 | TGTGATGTGACGTAGTGGTAC | 1.115 | 148.99 | 9.25 | 2.66 |
| aal-miR-993 | TACCCTGTAGTTCCGGGCTTTT | 1.106 | 0.00 | 0.00 | 0.00 |
| aal-miR-993-3p | GAAGCTCGTCTCTACAGGTAT | 1.106 | 0.00 | 0.01 | 0.00 |
| aal-miR-996 | TGACTAGATTACATGCTCGTCT | 1.437 | 328.42 | 40.97 | 77.02 |
| aal-miR-998-3p | TAGCACCATGAGATTCAGCTC | 1.744 | 74.26 | 13.77 | 56.91 |
| aal-miR-998-5p | ACTGAACTCTCGTGGGTCTGCA | 1.744 | 132.84 | 30.95 | 49.30 |
| aal-miR-999-3p | TGTTAACTGTAAGACTGTGTCT | 1.100 | 17.74 | 1.81 | 4.50 |
| aal-miR-9a-3p | TAAAGCTAGCATACCGAAGTTA | 1.517 | 4.32 | 0.90 | 2.09 |
| aal-miR-9a-5p | TCTTTGGTTATCTAGCTGTATGA | 1.517 | 46.70 | 2.16 | 4.63 |
| aal-miR-9b | TCTTTGGTGATTTTAGCTGTATGC | 1.785 | 11.16 | 1.88 | 4.00 |
| aal-miR-9c-3p | TAAAGCTTTAGTACCAGAGGTC | 1.785 | 128.67 | 52.17 | 85.65 |
| aal-miR-9c-5p | TCTTTGGTATTCTAGCTGTAGA | 1.785 | 38.67 | 5.01 | 11.29 |
| aal-miR-iab-4-3p | CGGTATACCTTCAGTATACGTA | 1.423 | 112.63 | 11.06 | 24.17 |
| aal-miR-iab-4-5p | ACGTATACTGAATGTATCCTGA | 1.423 | 5.14 | 0.90 | 2.41 |
| aal-miR-1174 | TCAGATCTAACTAATACCCAACT | 3.679 | 261.25 | 56.69 | 169.52 |
| aal-miR-1174-3p | TGACGTCATGCTCGATTGGCTC | 1.125 | 0.57 | 0.31 | 0.26 |
| mir-122 | TGGAGTGTGACAATGGTGTTT |  |  |  |  |
| aal-miR-1273f | GGAGTTGCGAGTTGCAGAG | 1.790 | 70.35 | 334.91 | 374.44 |
| aal-miR-1357 | AGATTATGAGAGCTGAGGGCA | 1.677 | 17.33 | 107.19 | 113.82 |
| aal-miR-1413 | GCCGCGAGGTGGGGCTTACTG | 1.156 | 12.86 | 115.32 | 80.57 |
| aal-miR-241-5p | GGAGGTAGTGACGAGAAATAA | 1.836 | 289.64 | 3,843.54 | 3,398.76 |
| aal-miR-2478 | CGTATCCCACTTCTGACACCA | 1.943 | 7.41 | 22.54 | 22.65 |
| aal-miR-2951-5p | AAGAGCTCAGCACGCAGGGGCGA | 1.836 | 744.06 | 6,699.51 | 4,446.20 |
| aal-miR-3811e-5p | TTGGCTTCCTTGCGGTGCACA | 1.108 | 3.7028 | 40.1339 | 183.667 |
| mir-3888-5p | GTGCTCTGAATGTCAACGT |  |  |  |  |
| aal-miR-6492 | GGATGACTGAAGCGGAGGAGG | 1.120 | 586.22 | 5348.38 | 7,955.86 |
| aal-miR-716b | ACAGATCTTGGTGGTAGTAGC | 1.836 | 142.15 | 1,222.59 | 752.24 |
| aal-miR-956-3p | TTTCGAGACCACTGCAAATCATT | 1.245 | 92,217.14 | 5,551.91 | 10,404.88 |
| aal-miR-956-5p | GTTTGAAATGGTCTCGTTAACT | 1.245 | 14.71 | 3.90 | 3.55 |
| aal-miR-1767 | AGACAGGAGAACAGCAAGGT | 1.874 | 85.37 | 358.14 | 1373.98 |
| aal-miR-4448 | GGCTCGTTGGTCTAGGGGT | 1.173 | 929.32 | 2,988.27 | 255.71 |
| aal-miR-4728-5p | TGGGAGGGCAGAGGGGCAGCA | 1.714 | 5.61 | 30.88 | 159.81 |
| aal-miR-6134 | TGAGGTAGGAAGAAATGTAGA | 1.150 | 3.86 | 54.05 | 238.35 |
| aal-miR-622 | AGAGTTCACTGGGTTGGAGGC | 1.2094 | 771.93 | 968.99 | 6,396.11 |
| aal-miR-6668-3p | TGGGATGTGGAATAGATTGGG | 1.878 | 1.868.98 | 451.28 | 3,563.37 |

^a^Reads by high throughput deep sequencing

C: Midguts from mosquitoes ingested with sugar solution

B: Midguts from mosquitoes ingested with regular blood meal

D: Midguts from mosquitoes ingested with artificial DENV-2 blood meal
